# Trichostatin A accentuates doxorubicin-induced hypertrophy in cardiac myocytes

**DOI:** 10.18632/aging.100203

**Published:** 2010-09-17

**Authors:** Tom C Karagiannis, Ann JE Lin, Katherine Ververis, Lisa Chang, Michelle M Tang, Jun Okabe, Assam El-Osta

**Affiliations:** ^1^ Epigenomic Medicine, Baker IDI Heart and Diabetes Institute, The Alfred Medical Research and Education Precinct, Melbourne, Victoria, Australia; ^2^ Department of Pathology, The University of Melbourne, Parkville, Victoria, Australia; ^3^ Epigenetics in Human Health and Disease, Baker IDI Heart and Diabetes Institute, The Alfred Medical Research and Education Precinct, Melbourne, Victoria, Australia; ^4^ Department of Anatomy and Cell Biology, The University of Melbourne, Victoria, Australia; ^5^ Faculty of Medicine, Monash University, Victoria, Australia

**Keywords:** Cardiac hypertrophy, doxorubicin, cardiomyocyte, cardiac differentiation, histone deacetylase inhibitor, Trichostatin A

## Abstract

Histone deacetylase inhibitors represent a new class of anticancer therapeutics and the expectation is that they will be most effective when used in combination with conventional cancer therapies, such as the anthracycline, doxorubicin. The dose-limiting side effect of doxorubicin is severe cardiotoxicity and evaluation of the effects of combinations of the anthracycline with histone deacetylase inhibitors in relevant models is important. We used a well-established *in vitro* model of doxorubicin-induced hypertrophy to examine the effects of the prototypical histone deacetylase inhibitor, Trichostatin A. Our findings indicate that doxorubicin modulates the expression of the hypertrophy-associated genes, ventricular myosin light chain-2, the alpha isoform of myosin heavy chain and atrial natriuretic peptide, an effect which is augmented by Trichostatin A. Furthermore, we show that Trichostatin A amplifies doxorubicin-induced DNA double strand breaks, as assessed by γH2AX formation. More generally, our findings highlight the importance of investigating potential side effects that may be associated with emerging combination therapies for cancer.

## INTRODUCTION

Conventional cancer therapies involve the use of combinations of surgery, radiotherapy and various chemotherapeutic regimens. The anthracyclines represent an extremely effective class of chemo-therapeutics which are used for the treatment of numerous haematological and solid malignancies [1]. They have been shown to induce cancer cell-death by a number of mechanisms including DNA binding and intercalation, generation of free radicals, inhibition of the topoisomerase II enzyme and damage to cell membranes [2-4]. In addition, anthracyclines have been shown to modulate various signalling pathways including those involved with apoptosis [5,6]. The most potent and widely used anthracycline is doxorubicin, an analogue that has potent broad-spectrum antineoplastic activity and has been used a frontline cancer chemotherapeutic for several decades [7]. However, the clinical application of doxorubicin is limited by cumulative, dose-dependent cardiotoxicity [8]. For example, clinical trials have indicated that 7% of patients treated with doxorubicin experience a cardiac event with a cumulative dose of 150 mg/m^2^ and the proportion reaches 65% with a cumulative dose of 550 mg/m^2^[9]. The various cardiomyopathies, including cardiac hypertrophy, that are associated with doxorubicin treatment are well known and have been described extensively [10-13].

Histone deacetylase (HDAC) inhibitors represent a new class of anticancer therapeutics. The first clinical compound is the hydroxamic acid, suberoylanilide hydroxamic acid (SAHA; Vorinostat) which has been approved by the US FDA for the treatment of cutaneous T-cell lymphoma (CTCL) [14,15]. Further, several HDAC inhibitors are currently undergoing evaluation in clinical trials and encouraging antineoplastic effects at well-tolerated doses have been observed in both haematological and solid cancers [14,15]. The effects of HDAC inhibitors are due to numerous mechanisms including, induction of differentiation, cell cycle arrest, production of reactive oxygen species, altered cell migration, mitotic and autophagic cell death, and induction apoptosis in cancer cell-lines in culture and *in vivo* [16-19]. Studies have shown that the cytotoxic and more recently the DNA damaging effects of HDAC inhibitors is much more pronounced in malignant or transformed cells compared to normal cell lines [20].

For cancer therapy, it is expected that HDAC inhibitors will be particularly useful when used in combination with conventional therapeutics [16,21-23]. Indeed, combinations of various HDAC inhibitors with radiotherapy or chemotherapeutics, including doxorubicin, have been widely investigated and synergistic or at least additive effects have been observed [24-31]. Given this emerging therapeutic strategy, it is important to evaluate the effects of combinations of HDAC inhibitors with conventional cytotoxic agents in relevant models to identify and investigate potential clinical side effects. Since cardiomyopathy is the most severe side effect of doxorubicin treatment, we evaluated the effects of combinations of the anthracycline with Trichostatin A, the prototypical broad-spectrum HDAC inhibitor, in cardiomyocytes [32]. An established cell culture approach of doxorubicin-induced cardiac hypertrophy in rat H9c2 ventricular myocardial cells was used as a model system [33]. Firstly, we evaluated the effects of doxorubicin on the expression of the hypertrophy-associated genes, ventricular myosin light chain-2 (MLC-2v), the alpha isoform of myosin heavy chain (α-MHC) and atrial natriuretic peptide (ANP) [34-38]. The effects of Trichostatin A on doxorubicin-induced hypertrophic responses in H9c2 cells were then examined.

## RESULTS

### H9c2 myoblasts differentiate to cardiac myocytes in the presence of retinoic acid

**Figure 1.**
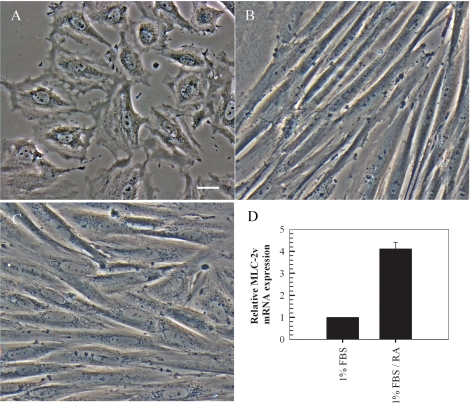
Retinoic acid induces differentiation of embryonic H9c2 myoblasts to cardiomyocytes in low serum supplemented with retinoic acid. Rat embryonic heart-derived myoblasts maintained in DMEM containing 10% FBS (**A**) were cultured in DMEM containing 1% FBS for seven days resulting in differentiation to skeletal muscle (**B**). A seven day culture in low serum media supplemented with 10 nM retinoic acid resulted in differentiation into cardiac myocytes (**C**). RT-PCR quantitation of MLC-2v transcripts (which display absolute cardiac tissue specificity) indicates overexpression of the gene in retinoic acid treated cells compared to cells cultured in low serum without retinoic acid (mean ± standard deviations of triplicates from a representative experiment, total of three independent experiments, (**D**). Bar = 5 μm; *x* 20 magnification.

It is well-established that chronic culture in low serum media containing all*-trans*-retinoic acid prevents transdifferentiation of embryonic cardiac H9c2 cells into skeletal muscle [39]. We confirmed the phenotype of embryonic myoblasts and monitored myogenesis and cardiac myocyte formation by phase contrast microscopy and RT-PCR (Figure 1). Freshly plated cells maintained in media containing 10% FBS were strictly mononucleated myoblasts that transdifferentiated into skeletal muscle cells, as observed by the parallel and regular elongated bundles, following a seven day culture in media containing 1% FBS (Figures 1A and 1B). Culture of H9c2 myoblasts in low serum media and stimulation with 10 nM all*-trans*-retinoic acid for seven days resulted in the maintenance of the cardiac phenotype with the elongated cells connecting at irregular angles (Figure 1C). Further confirmation of the cardiac phenotype was obtained by investigating the relative expression levels of MLC-2v, a gene which displays absolute cardiac tissue specificity [40]. The RT-PCR findings indicate a four-fold increase in the expression of MLC-2v in cells cultured in low serum media containing 10 nM all*-trans*-retinoic acid compared to cells cultured in low serum media without retinoic acid (Figure 1D).

### Doxorubucin induces a hypertrophic response in H9c2 cardiac myocytes

**Figure 2.**
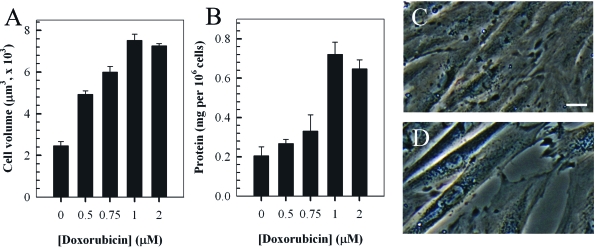
Doxorubicin (Dox) induces a dose-dependent hypertrophic response in rat H9c2 cardiomyocytes. Cells were treated with the indicated concentrations of doxorubicin for two hrs and cultured in fresh media for a further 24 hours prior to quantitation of cell volume (**A**) and total protein content per cell (**B**). Phase-contrast images of control untreated H9c2 myocytes (C) compared to cells treated with 1 μM doxorubicin (2 hr treatment followed by 24 hour incubation in fresh media, D). Bar = 5 μm; *x* 20 magnification.

The dose-dependent hypertrophic response in H9c2 cells induced by treatment with doxorubicin was examined by measuring the cell volume and total protein content (Figures 2A and 2B). Experiments involved incubating cells with various concentrations (0-2 μM) of doxorubicin for two hours followed a further incubation in fresh media for a further 24 hours. The findings indicated a dose-dependent increase in cell volume and total protein content up to a concentration of 1 μM doxorubicin. The change in phenotype and enlargement of H9c2 cardiomyocytes treated with 1 μM doxorubicin is also evident by phase-contrast microscopy (Figures 2C and 2D).

### Doxorubicin modulates the expression of hypertrophy-associated genes in cardiomyocytes

**Figure 3.**
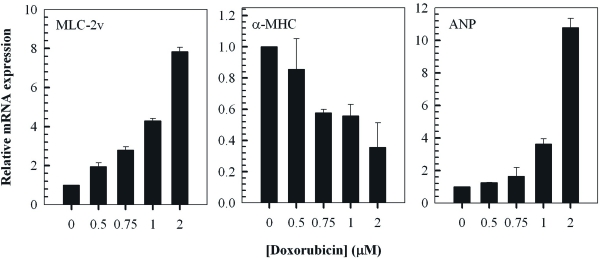
Doxorubicin modulates the expression of cardiac hypertrophy-associated genes in H9c2 myocytes. QT-PCR analysis of MLC-2v, α-MHC and ANP transcripts in cells treated with the indicated concentrations of doxorubicin for 2 hours followed by a 24 hour treatment in fresh media. Fold change of mRNA expression shown relative to untreated control H9c2 cells; mean ± standard deviations of triplicates from a representative experiment (total of three independent experiments) are indicated.

To further characterise the response of H9c2 cells to treatment with doxorubicin, the relative expression levels of well known cardiac hypertrophy-associated genes, namely MLC-2v, α-MHC and ANP, was examined by RT-PCR (Figure 3). The findings indicated a dose-dependent increase in the expression of MLC-2v and ANP which are known to be upregulated in cardiac hypertrophy [34-36]. Furthermore, doxorubicin induced a dose-dependent decrease in the relative expression of α-MHC representing another established hallmark of cardiac hypertrophy [34,37,38].

### Trichostatin A augments doxorubicin-induced hypertrophy in H9c2 cardiac myocytes

**Figure 4.**
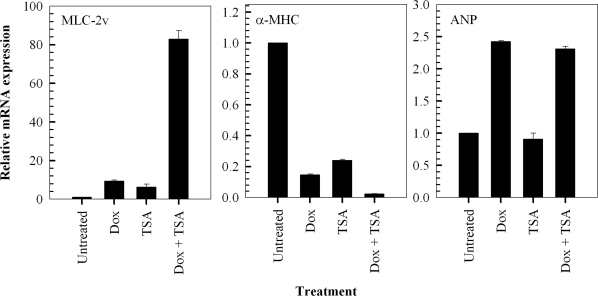
Trichostatin A (TSA) potentiates doxorubicin-induced hypertrophic responses in H9c2 cardiomyocytes by modulating the expression of hypertrophy-associated genes. QT-PCR analysis of MLC-2v, α-MHC and ANP transcripts in cells treated with 1 μM doxorubicin (Dox) for 2 hours followed by a 24 hour treatment in fresh media in the presence and absence of 1 μM TSA. Fold change of mRNA expression shown relative to untreated control H9c2 cells; mean ± standard deviations of triplicates from a representative experiment (total of three independent experiments) are indicated.

We investigated the effects of the prototypical and potent, broad-spectrum histone deacetylase inhibitor, Trichostatin A, on doxorubicin-induced hypertrophic responses in H9c2 cells. Firstly, we investigated the effects of a 24 hour exposure to 1 μM Trichostatin A alone, on the relative expression levels of MLC-2v, α-MHC and ANP. The findings indicate that Trichostatin A alone causes a significant modulation of MLC-2v and α-MHC gene expression, inducing a hypertrophic response in H9c2 cells (Figure 4). In addition, exposure of the cells to a combination of Trichostatin A and doxorubicin results in a greater increase in the relative expression of the MLC-2v and a greater decrease in the expression of α-MHC, compared to treatment with either compound alone (Figure 4). In contrast, exposure of the cells to 1 μM Trichostatin A for 24 hours does not result in the modulation of the ANP gene. Furthermore, the results indicate that Trichostatin A does not affect the significant increase in ANP expression observed when cells are exposed to 1 μM doxorubicin alone (Figure 4).

### Trichostatin A enhances doxorubin-induced DNA damage in cardiomyocytes

**Figure 5.**
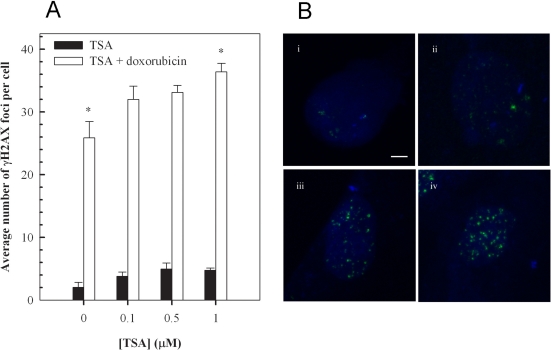
Trichostatin A (TSA) augments doxorubicin-induced accumulation of γH2AX foci in H9c2 cardiomyoctes. Cells pretreated with the indicated concentration of TSA for 24 hours were exposed to 1 μM doxorubicin for 1 hour, followed by a 24 hour treatment in fresh media. Cells were then stained for γH2AX foci, images were acquired with a Zeiss LSM 510 Meta Confocal microscope using 0.5 μm Z-sectioning and foci were quantitated using Metamorph (**A**). Mean ± standard deviations from two independent experiments (total of five independent experiments) are indicated (*p<0.001). Immunofluorescence visualization of γH2AX foci (**B**) in untreated H9c2 cells (i), cells treated with 1 μM TSA (ii), cells treated with 1 μM doxorubicin (iii) and cells treated with a combination of TSA and doxorubicin (iv) as described above. Bar = 2 μm; *x* 63 magnification.

Having established that the inhibition of histone deacetylase increases doxorubisin-induced hypertrophy, we determined whether the effect of TSA on cardiomyocytes conferred changes in DNA damage. Phosphorylation of the histone variant, H2AX on Ser-139 forming γH2AX, is a sensitive and reliable marker of DNA double-strand breaks [41,42]. Therefore, we utilised this phosphorylation event to evaluate the effects of Trichostatin A on doxorubicin-induced DNA damage (Figure 5). Quantitation of γH2AX foci indicated that a one hour incubation with 1 μM doxorubicin, followed by 24 hour incubation in fresh media, results in the formation of a significant number of DNA double-strand breaks - approximately 26 foci per cell compared to an average of approximately 2 foci per cell in untreated cells (Figures 5A, 5Bi and 5Biii). The findings also indicate a modest dose-dependent increase in γH2AX foci following 24 hour incubation with Trichostatin A (Figure 5A). Importantly, the results show a significant enhancement of doxorubicin-induced foci by Trichostatin A, particularly at 1 μM, highlighting a further mechanism by which the histone deacetylase inhibitor may augment doxorubicin-induced cardiotoxicity (Figures 5A and 5B).

## DISCUSSION

Our findings indicate that Trichostatin A augments doxorubicin-induced cardiac hypertrophy and DNA double-strand induction in H9c2 cardiac myocytes. We used clonal embryonic ventricular H9c2 myoblasts that have been isolated and established by selective passaging from BDIX rat heart tissue for our investigation [43]. This cell line is used widely given the ease of manipulation of the cells and the ability to maintain the cardiac phenotype in the presence retinoic acid has been well defined [39]. In accordance, with previous studies, we confirmed maintenance of the cardiac phenotype in the presence of retinoic acid and examined transdifferentiation of H9c2 myoblasts to skeletal muscle, following culture in low serum media without retinoic acid [39]. Furthermore, doxorubicin-induced cardiac hypertrophy is well-characterized in H9c2 cells, which is of importance for our investigations [33,44].

Cardiac hypertrophy, defined as an increase in cardiomyocyte size, is an adaptive response to a number of intrinsic (e.g. mutations of sarcomeric contractile proteins in familial hypertrophic cardiomyopathy) and extrinsic stimuli (e.g. hypertension). It is characterized by increased protein synthesis, sarcomeric reorganization and re-expression of fetal regulatory genes. Prolonged pathological cardiac hypertrophy is a major cardiovascular endpoint and is strongly associated with arrhythmias, heart failure and sudden death. Doxorubicin is thought to induce cardiac hypertrophy, a dose-limiting side effect, by the formation of free-radicals and lipid peroxidation [45]. This has lead to numerous investigations into the potential of free radical scavengers to ameliorate the effects of doxorubicin and more recently liposomal and nanoparticle-based formulations of the drug have been prepared [46-50]. In this context, an important link between cell growth, senescence and hypertrophy has been suggested and it has been previously shown that doxorubicin-induced hypetrophy and senescence can be inhibited by the immunosupresant, rapamycin [51-53].

In H92 cells, doxorubicin has been shown to induce a hypertrophic response and this has been associated with increased protein content and with morphological changes which have been correlated with increases in cell size and apoptosis [33,44]. Therefore, these cells have been used extensively to investigate doxorubicin-induced cardiotoxicity and to evaluate the effectiveness of protective compounds including, carvedilol, rosmarinic acid and thromobopoietin [54-57]. Our findings, are similar to those previously published and indicate a dose-dependent increase in cell size and protein in H9c2 myocytes following treatment with doxorubicin (Figure 2) [33,44]. In addition, there has been an increase in our understanding of molecular pathways associated with doxorubicin-induced cardiotoxicity in H9c2 cells. Since reinduction of the fetal cardiac gene program is known to be a hallmark of cardiac hypertrophy, we investigated the effects of doxorubicin on expression of the MLC-2v, α-MHC and ANP genes [58]. These serve as well-established markers of cardiac hypertrophy [34-38,58]. In accordance with our expectations the findings indicate that a dose-dependent doxorubicin-induced increase in MLC-2v and ANP expression and a decrease in α-MHC expression (Figure 3). The result for ANP is consistent with a previous findings indicating that doxorubicin causes an induction of the hypertrophic markers, ANP and beta natriuretic peptide [59].

The antineoplastic properties of HDAC inhibitors are caused, at least in part, by the accumulation of acetylated nuclear core histones resulting in the altered transcription of a small number of genes, some of which important in regulating cellular proliferation, cell cycle progression and apoptosis [22,23]. Moreover, the effects of HDAC inhibitors in malignant or transformed cells can be attributed to altered activity of numerous critical proteins including transcription factors and key regulators of signaling cascades [14,15]. Furthermore, it is relatively well-established that HDAC inhibitors augment the cytotoxic effects of ionising radiation and chemotherapeutics, including doxorubicin, in cancer cells in culture and *in vivo* [24-31,60,61]. However, the effects of HDAC inhibitors in normal cells, and more specifically in cardiac hypertrophy, require clarification. For example, it has been shown that class II HDAC enzymes suppress cardiac hypertrophy and this has been associated with repression of the activity of myocyte enhancer factor 2 [62]. However, a number of studies have indicated a favorable role for HDAC inhibitors, particularly Trichostatin A, in cardiac hypertrophy both *in vitro* and *in vivo*[63-65]. Of particular importance, a previous study has indicated that Trichostatin A inhibited agonist-induced hypertrophy in neonatal rat ventricular myocytes and this effect was correlated with histone hyperacetylation and inhibition of fetal gene expression including α-MHC [63]. However, in that particular study a relatively low concentration of Trichostatin A (85 nM) was investigated. Our findings using 1 μM Trichostatin A indicate that the HDAC inhibitor alone induces a pro-hypertrophic response and amplifies doxorubicin-induced hypertrophy (Figure 4). We chose to investigate the higher concentration, which we have already determined is known to induce robust histone hyperacetylation and alterations in gene transcription as well as cell-death in malignant cells [26,27,66].

In addition to altering cellular morphology and modulating signaling pathways, doxorubicin has been shown to induce DNA damage and induction of p53 in H9c2 cells [67-69]. We investigated the effects of Trichostatin A on doxorubicin-induced DNA double-strand breaks using γH2AX as a molecular marker. Our findings indicate that pre-treatment of H9c2 cells with Trichostatin A potentiates doxorubicin mediated DNA damage, even at a relatively low concentrations of the HDAC inhibitor (100 nM, Figure 5). Therefore, augmentation of initial DNA damage represents an additional mechanism by which HDAC inhibitors may enhance the cytotoxicity of conventional antineoplastic chemotherapeutics.

Overall, histone deacetylase inhibitors have emerged as a new class of anticancer therapeutics, which are anticipated to be most effective when used in combination with conventional cancer therapies. Although they have been shown to induce cell-death and apoptosis preferentially in cancer and transformed cells compared to normal cells, the effects of combinations of HDAC inhibitors with other cytotoxic agents in normal cells have not been well-investigated. Our current findings which indicate that Trichostatin A potentiates doxorubicin-induced cardiac hypertrophy and DNA damage highlight the need for further investigation of potential side effects associated with new combination therapies for cancer.

## MATERIALS AND METHODS

### Cell culture, differentiation and treatment

The rat embryonic ventricular myocardial, H9c2 cells were obtained from the American Type Culture Collection (Manassas, VA, USA) and were grown as monolayers in Dulbecco's modified Eagle's medium (DMEM), containing 10% fetal bovine serum (FBS, In Vitro Technologies, Victoria, Australia), 100 U//ml penicillin and 100 μg/ml streptomycin (Invitrogen, Carlsbad, CA, USA), at 37C in a humidified atmosphere with 5%CO_2_. Prior to confluence (typically 60-70%), cells were passaged using 0.5% trypsin-EDTA (Invitrogen) and centrifugation (250 × g for 5 minutes) and seeded at ratios of 1:2 or 1:3 in DMEM containing 10% FBS for 24 or 48 hours. Cells were then cultured in DMEM containing 1% FBS with (to maintain the cardiac phenotype) or without (myogenic transdifferentiation) 10 nM all-*trans*-retinoic acid (Sigma-Aldrich, St. Luis, MO, USA) for 7 days and the culture media was changed daily.

The experiments described below, which required doxorubicin (Ebewe Pharma, Unterach, Austria) and Trichostatin A (Sigma-Aldrich) treatment, were performed with cells that were cultured in 1% FBS containing 10 nM all-*trans*-retinoic acid for 7 days. For experiments involving treatment with doxorubicin, cells were incubated with various concentrations (0-2 μM) of the anthracycline for 2 hours (except for the γH2AX immunofluorescence experiments described below; cells were incubated with doxorubicin for 1 hour). The cells were washed twice with phosphate buffered saline without calcium and magnesium and were incubated for a further 24 hours in fresh media. For relevant experiments, H9c2 cells were treated with various concentrations of Trichostatin A (0-1 μM) for 24 hours. Trichostatin A was added to the media immediately following the 2 hour incubation with doxorubicin, except for the γH2AX immunofluorescence experiments described below, which involved a 24-hour pre-treatment with the HDAC inhibitor prior to exposure to doxorubicin for 1 hour.

### Cell size and protein content

Adherent cells were detached using 0.05% trypsin EDTA and rounded cells were imaged using an Olympus (CKX41, Tokyo, Japan) microscope and × 20 lens. Cell diameters were measured using Image J (Fiji Version 1.44a) software and cell volume was calculated using the equation for the volume of a sphere (4/3 × π × radius^3^).

For determination of protein content, cells were collected by trypsinization and lysed with mammalian protein extraction reagent (Thermo Scientific, Rockford, IL, USA) and complete protease inhibitor cocktail (Roche, Indianapolis, IN, USA) at 4°C for 30 minutes. The suspension was centrifuged at 13,000 × g for 10 minutes at 4°C and the supernatant was collected. Protein concentration in the total cell lysates was measured at 595 nm using the Bradford assay with bovine serum albumin standards [70].

### Real-time polymerase chain reaction (RT-PCR)

RNA was extracted from H9c2 cardiomyocytes using Trizol reagent (Invitrogen) and DNA was removed using the Turbo DNA-free™ kit (Ambion Inc., Austin, TX, USA) according to the manufacturer's instructions. Total RNA (1 μg) was converted to cDNA using random primers and Moloney murine leukemia virus reverse transcriptase (Sigma-Aldrich). Primers were designed using Primer Express® Software v2.0: MLC-2v 5'- CCTAACGTCACCGGCAACC-3' and 5'- TTTGGTTCACATCATCACCCA-3; α-MHC, 5'-ACACGAAGCGTGTCATCCAG-3' and 5'-GGTCCCCTATGGCTGCAAT-3'; ANP, 5'- TCTTCCTCTTCCTGGCCTTTT-3' and 5'- CGGGATTTGCTCCAATATGG-3'; β-actin, 5'-CCTCTGAACCCTAAGGCG-3' and 5'-AGGGACAACACAGCCTGGAT-3' (Sigma-Aldrich). Fold changes (ΔCt) were calculated in the following manner: the cycle number (Ct) of the target genes were extrapolated using the software analysis program (SDS 1.9, Applied Biosystems) and was subtracted from the Ct of the input control. All means, standard deviations and statistics were calculated as a fold value.

### γH2AX immunofluorescence

The number of γH2AX foci in H9c2 cell nuclei following treatment with doxorubicin, Trichostatin A and combination of pretreatment with Trichostatin A followed by doxorubicin, were quantitated as described previously [70].
